# Public–private partnerships in practice: collaborating to improve health finance policy in Ghana and Kenya

**DOI:** 10.1093/heapol/czy053

**Published:** 2018-06-13

**Authors:** Lauren Suchman, Elizabeth Hart, Dominic Montagu

**Affiliations:** 1 Institute for Global Health Sciences, University of California San Francisco, 550 16th Street, 3rd Floor Box 1224, San Francisco, USA; 2 Department of Sociology University of California Davis, 1 Shields Avenue, Davis, USA

**Keywords:** Health financing, health policy, international health, non-governmental organizations, partnerships, public/private

## Abstract

Social health insurance (SHI), one mechanism for achieving universal health coverage, has become increasingly important in low- and middle-income countries (LMICs) as they work to achieve this goal. Although small private providers supply a significant proportion of healthcare in LMICs, integrating these providers into SHI systems is often challenging. Public–private partnerships in health are one way to address these challenges, but we know little about how these collaborations work, how effectively, and why. Drawing on semi-structured interviews conducted with National Health Insurance (NHI) officials in Kenya and Ghana, as well as with staff from several international NGOs (INGOs) representing social franchise networks that are partnering to increase private provider accreditation into the NHIs, this article examines one example of public–private collaboration in practice. We found that interviewees initially had incomplete knowledge about the potential for cross-sector synergy, but both sides were motivated to work together around shared goals and the potential for mutual benefit. The public–private relationship then evolved over time through regular face-to-face interactions, reciprocal feedback, and iterative workplan development. This process led to a collegial relationship that also has given small private providers more voice in the health system. In order to sustain this relationship, we recommend that both public and private sector representatives develop formalized protocols for working together, as well as less formal open channels for communication. Models for aggregating small private providers and delivering them to government programmes as a package have potential to facilitate public–private partnerships as well, but there is little evidence on how these models work in LMICs thus far.

**Key Messages**

- Shared goals, mutual understanding of what each sector can offer, and ongoing, structured communications are key factors in creating a successful public–private partnership.
- Developing joint cross-sector protocols for communication and internal protocols at the international NGOs (INGOs) will be important for sustaining successes and creating avenues for future work together.
- Aggregating franchised private providers to facilitate their interactions with government may be one way to encourage ongoing partnership, but this is an area for further inquiry in low- and middle-income countries.

## Introduction

Universal health coverage (UHC) is a key priority set out by both the World Health Organization and the United Nations General Assembly (World Health Assembly 2005; World Health Organization 2010). Social health insurance (SHI) schemes, one mechanism for achieving UHC, have therefore become increasingly important in low- and middle-income countries (LMICs) as they work to achieve this goal. In order to ensure comprehensive health insurance coverage for a broad population at reasonable cost, SHI schemes are generally designed such that individuals pay into a central fund, either indirectly through taxes or directly through wage-based contributions, and receive a set package of subsidized health services through accredited providers (Carrin and James 2004; World Health Assembly 2005; Savedoff *et al.* 2012). However, because certain populations cannot afford any financial contribution either through taxes or through direct payments, a number of countries, such as Indonesia (Aspinall 2014), have created hybrid SHI systems in which government monies are used to cover these populations.

Despite such efforts to extend their reach, LMICs often face challenges in achieving broad and sustained population coverage through their SHI schemes through public providers alone. Research suggests health system weaknesses among public providers, including inadequate staffing of health facilities (Ganle *et al.* 2016) and a lack of basic medicine and health supplies (Jenkins *et al.* 2013), as well as an insufficient number of public health facilities where beneficiaries can use their coverage, prompting some to discontinue their enrolment (Agyepong *et al.* 2016).

To address some of the challenges of SHI systems and ultimately achieve UHC in these countries, it is critical to meaningfully include private providers in these systems (Prata *et al.* 2005; Stallworthy *et al.* 2014; Morgan *et al.* 2016). Despite the fact that private providers make up a significant proportion of the provider landscape in a number of LMICs, we know little about their experiences with SHI systems, though research suggests these providers face very different challenges than public providers (Sieverding *et al.* 2018). Since private providers rarely interact with government systems to the extent that public providers do (Sieverding *et al.* 2018), it is challenging for them to give SHI officials the meaningful feedback that could result in these systems becoming friendlier to the private sector. Greater formal collaboration between the public and private health sectors is an obvious way to facilitate this feedback and, as a result, strengthen health systems.

This study provides input to address this need, focussing on the face-to-face interactions that facilitate the intersection of public financing and private provision, and which offer the greatest opportunity for positive synergies between government and private sectors. Using a case study from Ghana and Kenya, we track cross-sector collaboration over a 5-year period based on interviews with government officials in both countries, as well as several large international NGOs (INGOs) working together in a formal partnership.

The data for this study was collected through the qualitative evaluation of the African Health Markets for Equity (AHME) project. AHME is an initiative that uses National Health Insurance (NHI) to link supply (private providers) with demand (clients) in order to shift health markets toward providing quality healthcare to low-income patients in Kenya and Ghana. On the supply side, AHME guides private providers organized in a social franchise network^1^ through facility-level quality improvement, and also assists with NHI accreditation and re-accreditation. This work is supplemented by work directly with the NHIs that aims to make the accreditation process clearer and more efficient for private providers. Once accredited, AHME franchised providers can be paid by the NHIs for primary care and maternity services, depending on their facility type and the type of contract they receive. On the demand side, AHME works with government agencies to extend subsidized insurance coverage to those with lowest income and enrol them into NHI schemes. As part of this goal, AHME supports the NHI agencies in both countries with the implementation of pro-poor subsidy programmes.

Our findings indicate a significant shift in cross-sector understanding and willingness to collaborate in both countries, particularly on the public sector side. This was established through shared goals and perceived mutual benefit among both public and private sector partners, and was accomplished through ongoing, structured communications. We conclude with recommendations for future public-private partnerships (PPPs) focussing on public financing for healthcare in LMIC health systems.

### Public–private collaboration in health systems

Public–private collaborations in the provision of health services have a very long history in OECD countries and form the background of the Bismarckian models of healthcare that predominate across Europe (Paris *et al.*, 2010). A combination of strong government regulation and mixed-market care delivery in many wealthy countries has facilitated the provision of high-quality care in a sustained way (Cyrus Roeder and Yanick 2012). However, this experience is not easily replicated where regulatory or financial institutions are weaker, as in most LMICs. Indeed, it is common in LMICs to think about parallel healthcare financing and delivery systems, operating adjacent to one another, with some overlap in clinical providers but largely independent infrastructure, patient populations and financing. This stands in contrast to the integrated models in OECD countries, where the ownership and management of service providers may be either public or private, but the financing, regulation, and patient populations are largely indistinguishable (Busse *et al.* 2007). Moving from a separate-but-parallel ‘mixed’ health system to the ‘coordinated’ model common across most OECD countries and a few exceptional middle-income countries is critical to UHC. The importance of this shift is easily understood in theory, but has proven to be difficult in practice (Lagomarsino *et al.* 2009).

Alongside increased understanding of the importance of private provision in many LMICs, there has been growing attention over the past decade to identifying and encouraging models for public–private collaboration (Buse and Walt 2000). These kinds of partnerships, or PPPs [not to be confused with hospital or infrastructure PPPs, which focus on large-scale co-investment (Sekhri *et al.* 2011; Montagu and Harding 2012)], fall into five domains summarized by the World Bank as including: policy and dialogue; information exchange; regulation; financing; public provision of services (International Finance Corporation 2011). The most documented collaborations of this kind touch multiple domains and work with or through non-profit intermediaries, relying on ongoing interaction between public and private partners (Montagu *et al.* 2016). Given that getting public and private relationships ‘right’ will be important to achieving UHC, understanding these collaborations well, how they work, how effectively, and why, remains a new and imperfect field of study in which more information is needed (Morgan *et al.* 2016).

Although there are only a small number of previous studies on PPPs in the health sector in both Ghana and Kenya, findings from Ghana have suggested that NGOs could be valuable to government for their ability to increase reach and to offer technical expertise (Hushie 2016), although disruptions in funding and slow implementation from the public sector side proved challenging for these relationships (Amo-Adjei 2016). Similarly, one study of a multidisciplinary partnership to combat childhood cancer in Kenya suggested that consistency and flexibility are important to make public–private partnerships successful (Hill *et al.* 2016). However, while there is a more established literature of PPPs in the health sector in Kenya, much of the literature focuses on experiences among service providers on the ground (Chakaya *et al.* 2009; van de Vijver *et al.* 2013; Laktabai *et al.* 2017), or specifically on partnerships between government and global pharmaceutical companies (Vian *et al.* 2007), as opposed to the policy-level processes addressed in this article. One exception is Ravishankar *et al.* (2016), which examines synergy between government and private healthcare providers in the context of health financing in Kenya, India and Uganda. Through a literature review and supporting qualitative interviews, Ravishankar *et al.* (2016) identified five major challenges the public and private sectors in health face when working together: a lack of information sharing; weaknesses in management capacity; funding insecurity; mismatched organizational styles and differing priorities; and corruption.

### Context

Ghana’s National Health Insurance Scheme (NHIS) was established in 2003 and members have been accessing benefits since 2005. The NHIS aims to cover Ghana’s entire population with a single system of coverage (Lagomarsino *et al.* 2012) and all population groups receive the same benefits package (Otoo *et al.* 2014). Providers accredited with NHIS must offer a minimum package of services, but the broad package includes inpatient and outpatient care, emergency services, and maternity services (Witter and Garshong 2009; Saleh 2013; Otoo *et al.* 2014). Under NHIS, provider reimbursement operates on a fee-for-service model in which providers submit claims to NHIS for individual services rendered and should expect to receive payment within 4 weeks of submission (Witter and Garshong 2009). However, there is evidence that reimbursement delays are common (Agyepong and Nagai 2011; Sodzi-Tettey *et al.* 2012). As of 2013, Ghana’s NHI Agency estimated that 40% of accredited providers were from the private sector National Health Insurance Authority (NHIA 2013).

Although Kenya’s National Hospital Insurance Fund (NHIF), started in 1966, is technically much older than the Ghana NHIS, coverage under the NHIF has historically been much more limited. The scheme was designed to cover inpatient services for civil servants and formal sector employees (Abuya *et al.* 2015), both of whom still pay for coverage through payroll deductions, but it has expanded to include outpatient and maternity services (Githinji 2016; Netherlands Enterprise Agency 2016). Informal sector employees can also pay monthly for coverage (Abuya *et al.* 2015). Under this scheme, providers are paid differently depending on the types of services they offer; inpatient services are reimbursed on a fee-for-service model, while outpatient services operate on a capitation model (Okech and Lelegwe 2016). In a capitation system, providers are paid on a monthly basis depending on the number of clients registered to their clinic, regardless of how many clients access services. Estimates suggest that 40% of all health expenditures in Kenya are spent on private providers and that government financing makes up the largest proportion (34%) of all health spending (Netherlands Enterprise Agency 2016).

## Methods

The data for this study were collected through the qualitative evaluation of the AHME project. The AHME qualitative evaluation is divided into two components: one component broadly examines the supply (provider-focussed) and demand (client-focussed) aspects of the initiative; and the second component, a process evaluation, examines the evolution of the AHME partnership and its policy work. For the purposes of this paper we focus on one of the process evaluation’s sub-objectives: to examine how and why AHME has influenced the NHI systems’ perspectives on the integration of private providers into national health payment systems in Ghana and Kenya.

The data analysed below was collected through semi-structured, in-depth interviews with government officials, donors and representatives from the international organizations that made up the AHME partnership when the data were collected. The AHME partners include three INGOs: Marie Stopes International (MSI); Population Services International (PSI); and the PharmAccess Foundation (PharmAccess); and one intergovernmental organization, the International Finance Corporation (IFC). Interviewees were selected from organizations directly involved with the AHME project, because AHME provided a unique environment for collaboration between these organizations and the public sector.^2^ Measures were taken to increase data reliability and validity throughout the sampling, data collection, and analysis phases; these steps are detailed below.

### Sampling

Data for this study were collected during four rounds of data collection over a 5-year period. Data collection was conducted in: 2013, 2014, 2016 and 2017. During each round, UCSF and a local research partner, Innovations for Poverty Action, co-ordinated with the AHME implementation team to contact key staff from the AHME partner organizations in Ghana and Kenya, and invited them to participate in an interview. AHME partners at the global level were identified among members of the donor organizations and the AHME leadership team. Government officials were identified through referrals by interviewees and also were interviewed in country. We conducted few interviews with government counterparts because we sought to interview those most familiar with the project, and there were not many NHI representatives who worked directly with AHME.

Each stage of the process evaluation focussed on a slightly different set of questions in order to respond to the changing nature of the AHME initiative over time. As such, we used purposeful criterion sampling combined with opportunistic sampling (Palinkas *et al.* 2015) at each stage of data collection to best respond to questions posed at different points in the process evaluation. Interviewees were selected based on the degree of their involvement with the AHME partnership and particularly with specific activities under investigation at different points in the evaluation. For example, in order to answer questions regarding AHME’s influence on government views of private providers, we interviewed AHME partners who had worked directly with the respective NHIs, as well as NHI officials who had participated in collaborative activities with the partnership. Depending on the extent of their involvement with the AHME partnership, some participants were only interviewed once while others participated in several rounds.

### Data collection

The research team conducted 87 interviews with 62 interviewees from 2013 to 2017. Data were collected in Ghana in July 2013, September 2014, June 2016 and February 2017. Data were collected in Kenya in November 2013, September 2014, June 2016 and 2017. In interviews, global partners were asked about their perceptions of the relationships the AHME partners were forming with government over time. Representatives from the AHME partnership were asked about their experiences working with the NHIs: how these relationships formed and developed over time; who was involved and in what way; and what they expected the relationships to look like in the future. Officials from the NHIs were asked complementary questions about their collaborations with AHME: how they perceived the role of private providers in the respective NHI systems; how the collaboration with AHME began and how it evolved over time; the extent to which NHI goals aligned with AHME’s goals; and their hopes for the future of collaborative work with the partnership. Most interviews were conducted in-person, while some were conducted over the phone or via Skype in cases where the interviewee was not based in-country (e.g. AHME leadership based in London). Interviews lasted approximately one hour (Table 1).^3^

**Table 1.** Sample size per data collection round

	Global Partners^a^	In-country Partners^b^		Government Representatives^c^	
		Kenya	Ghana	Kenya	Ghana
Round 1 (2013)	4	11	6	0	0
Round 2 (2014)	1	12	4	2	0
Round 3 (2016)	16	7	4	2	4
Round 4 (2017)	2	7	4	4	2

a AHME partners affiliated with one of the implementing INGOs who are not based in Kenya or Ghana.

b AHME partners affiliated with one of the implementing INGOs who are based in Kenya or Ghana.

c Officials working with the Kenya NHIF or Ghana NHIS who work directly with AHME.

### Data processing and analysis

All interviews were digitally recorded by the authors, except in a small number of cases where the interviewee declined to be recorded and the interviewer documented the conversation through notes. Recorded interviews were transcribed by a team of professional transcriptionists. A select sample of interviews was back-checked by the UCSF programme manager to ensure accuracy.

The authors coded the transcripts in Atlas.ti using an open-coding approach, in which codes and sub-codes were derived from the data rather than pre-determined. We adopted an iterative approach to developing the codebook in which codes and sub-codes were refined over the course of the coding process as each interview was incorporated. Code families spanned global and in-country implementing partners; and government interviewees across countries. The analysis process indicated that data saturation was reached for both the implementing and global partners, and government samples in both countries.

## Results

Initially, interviewees had incomplete knowledge about the potential for synergy between the public and private health sectors in Kenya and Ghana. We find that motivations for a cross-sector collaboration originated from shared goals and the potential for both parties to benefit: the need for technical support, from the perspective of the NHI officials, and the need for institutional infrastructure, from the AHME partners. The nature and character of the relationship was shaped by frequent face-to-face interactions and reciprocal feedback. This process led to a collegial, mutually beneficial relationship between AHME partners and their NHI counterparts.

### The public–private relationship at the start of AHME

When AHME began, the NHIs had a history of working with private providers. However, particularly in Kenya, this work was mostly limited to large urban hospitals, as opposed to the small private providers involved in AHME. As a result, the NHIs had little understanding of what they could gain from working with these providers. Meanwhile, private sector partners perceived suspicion from the public sector side and were unsure if the risks of greater scrutiny and regulation that could accompany closer linkages to the government would have offsetting benefits. One interviewee, an AHME implementing partner in Ghana, suggested that NHI staff perceived the private sector to be ‘self-seeking’, as opposed to the ‘good-seeking’ public sector. Similarly, another partner in Ghana noted:

*The public sector thinks that the private sector are merchants, they are only profiteering, they are only looking for profits…The public sector thinks that they are socially minded but the private is commercially minded…* (AHME Implementing Partner, Ghana).

This interviewee suggested that the perceived opposing goals of the public sector as ‘socially minded’ and the public sector as ‘commercially minded’ could impede their ability to successfully collaborate.

In many OECD countries, the public and private sectors are understood to be collaborating in serving and improving public health through: public financing for private delivery of care to the overall population; shared information, training, and data; cross-referrals; and shared technology, labs, and blood banks. This perspective is rare in Ghana and Kenya where the public and private sectors are often understood to have fundamentally different goals and approaches. In fact, some interviewees thought that the two sectors were in competition with one another. Since clients can choose freely between public and private healthcare facilities, one interviewee suggested, the public sector may be hesitant to collaborate with private providers if they believe that these providers are taking potential clients away from public facilities.

Conversely, Kenyan NHIF officials interviewed in Round 2 (2014) of data collection^4^ suggested that they simply did not know very much about small private providers, having engaged minimally with them in the past. This lack of engagement came from structural issues that made it difficult for these providers to engage with the NHIs in the first place. One such issue was the NHIF policy that only awarded contracts for inpatient services. This policy, by default, excluded most small private providers who were only able to offer outpatient coverage. As one NHIF official pointed out:

*You find that from our accreditation standards, [small private providers] were locked out even before we started the outpatient [services] because they would largely provide primary health care services. This is because the standard is so stringent because it wants to qualify you to provide services to our members, and therefore it’s not just anyone is allowed. And secondly since it was just aiming for in-patient* (NHIF Official, Kenya).

Stringent public-sector policies, intended to insure quality services, therefore precluded private clinics from gaining accreditation and developing deeper relationships with government. Similarly, another official recognized that, while they had relationships with some AHME partners (e.g. the IFC and PharmAccess) at the policy level, they had not previously worked with any of the INGOs overseeing the franchised clinics. When asked why the public and private sectors didn’t collaborate more, an AHME implementing partner in Kenya noted,

*I think it’s just because of lack of information [within the public sector] on what the private sector could do.*

### Motivations for public–private collaboration

*And I think that for us as NHIA our motivation for joining [AHME] is the fact that* ***it’s coming to help us achieve our core mandate*** *. And the core mandate of NHIA is to ensure…universal health coverage for members, and to achieve that universal health coverage you need to cover the poor. If you neglect the poor there is no way we can achieve universal health coverage. And so it comes in handy to ensure that our core mandate of universal health coverage is achieved. And that’s why we should support [AHME] to make it happen (NHIA Official, Ghana)*.

Although the AHME partners perceived tensions between the public and private sectors at the beginning of the programme, shared goals between AHME and the NHIs in both Kenya and Ghana motivated both sides to work together. Since the AHME model uses NHI as a mechanism to connect healthcare supply and demand for low-income populations, the partners had to reach out to officials working in the respective NHI offices to realize the programme’s ultimate goal. In other words, the NHIs provided the overall infrastructure within which the AHME model functioned. From the perspective of the NHI officials, our data indicate that the NHIs lacked the capacity in some areas to achieve their mandate to provide health coverage to all citizens. Thus, the public sector hoped to benefit from the technical support and financial resources AHME offered.

Indeed, interviewees from both the AHME partners and the NHIs recognized that the public sector faced challenges and could benefit from the assistance offered by the INGOs that represented the private sector. Further, the AHME partners approached the NHIs and offered assistance for free, making it easy for the government officials to accept the offer to work together. One interviewee described how the public sector needed help with the issue of assuring consistent quality across health clinics:

*Interviewer: Do they see added value with working with private provider networks?*

*Respondent: They do. In fact, they even told us “we really want you to discuss how you can help us with quality issues”* (AHME Implementing Partner, Kenya).

AHME partners that were working with the private sector complemented existing public-sector programmes by providing their expertise in areas like quality assurance, marketing and community outreach. Thus, the public sector came to view the AHME partner organizations as a resource to help them solve their problems.

*I will say [NHIS representatives] are very receptive, they are ready to roll, because they know they have problems. They acknowledge those bottlenecks…They’re receptive in designing solutions with us to solve the problem. We just had a meeting with them, they are good, receptive, and they are ready to work with AHME in exploring these solutions* (AHME Implementing Partner, Ghana).

In addition to the expertise of the organizations representing the private sector, the broad reach of private sector providers was appealing to the NHI agencies. According to one AHME implementer in Kenya, the NHIF was aware that their public facilities would not adequately reach poor populations, which piqued their interest in collaborating with AHME. Indeed, government officials came to see private providers as another means to achieve their goal of UHC by filling important gaps in their ability to serve both rural and poor patients, including through the spread of access to NHI-accredited facilities.

*I think the value is there because, for NHIF…you want to have a network of facilities that are across everywhere, so that you are not limiting access to services for the members. And they also have facilities where they can select services, because it works both ways. If there are no [NHIF-accredited] providers where I live, I will not pay for NHIF because it doesn’t make sense. So I think it’s really demand driven* (Former NHIF Official, Kenya).

Further, public sector representatives discussed the benefits of working specifically with franchised private providers. As officials from both the NHIF in Kenya and the NHIA in Ghana noted, partnering with social franchise networks was especially valuable to the NHIs because the networks ensure a baseline level of quality for private clinics.

*At least you know there are these providers where the quality is being checked. Then I think it adds value to NHIF definitely knowing that you have a range of providers that are already checked, and you don’t have to put so much effort in that area in terms of quality monitoring* (Former NHIF Official, Kenya).

Recognizing the benefits of working with private providers, one interviewee at the NHIF went so far as to call private sector facilities ‘saviors’ of the public sector, specifically noting that franchise networks can increase access for rural beneficiaries. Although social franchise networks are typically concentrated in urban areas (Viswanathan and Seefeld 2015), AHME is making concerted efforts to expand into underserved rural areas. Through working with AHME, the NHI officials were therefore able to see the concrete ways in which partnering with private clinics could help them achieve their mission.

However, some interviewees suggested that the NHIs’ desire to work with the private sector was merely demand driven, rather than being motivated by the added value of private facilities or social franchise networks in particular. Several respondents argued that the public sector simply would not be able to meet increasing demand for health services without private providers. As one AHME partner suggested, the government had ‘no option’ but to collaborate with the private sector, because a significant proportion of their beneficiaries rely on the private sector for their healthcare:

***The government has no option*** *. Some of the research that has been done has shown that 55% of all health services consumed by Ghanaians are provided by the private sector… . So it gets to a point where the government has no option but to bring the private sector onboard and I think government recognizes that* (AHME Implementing Partner, Ghana).

### Development of the public/private relationship

The public sector expressed some motivation or need to partner with the private sector, but the nature and timeline of that collaboration were particularly important for the evolution of their perspective about private sector healthcare. One AHME partner, the IFC, was initially brought into the partnership because they had pre-existing relationships with government. As another AHME partner in Kenya suggested, the IFC was a critical entry point to establish a relationship between AHME and the NHIs:

*But one of the things I think AHME did well was the IFC bit, the policy bit of putting a partner who is respected by the government to try and make the change, policy issues and decisions…it will really influence how AHME looks like in future because you are influencing decisions at a very high level, not just say it impacts all of us as implementers. So, if we really focus on the good policy issues the project definitely thrives* (AHME Implementing Partner, Kenya).

Although the IFC initially acted as an intermediary between the NHIs and the other AHME partners, these partners formed a relationship with their public sector counterparts by working on the design and implementation of several pro-poor programmes that were designed in collaboration with government: the Health Insurance Subsidy Programme (HISP; targeting low-income households) and Supa Cover program (targeted to informal sector workers) in Kenya and the pilot of a Common Targeting Mechanism tool for identifying poor populations in Ghana.

Developing the public/private relationship was a lengthy process that involved research, assessments, writing and presenting reports and regular communication between the leadership of the AHME consortium and various government agencies. As one interviewee recalled, developing the terms of their collaboration required a number of in-person meetings with officials across several levels of government:

*We’ve had about six or seven different meetings… … that’s formally sitting down to negotiate the DSF [demand-side financing] program. And we had several meetings just sitting down and talking about what needs to be done. I’ve met with the Chief Executives on a one-on-one basis, and by chief executives I mean all of them: The minister, the Director General of the Ghana Health Service, The Chief Executive of the NHIA, and then the presidents of the associations. We’ve met with parliament thrice as a body, sitting down and discussing what should be done, presented the evidence that we have* (AHME Implementing Partner, Ghana).

Another interviewee described the iterative process of researching options for collaboration and finally developing detailed work plans that prompted the need for multiple meetings:

*Then the teams went back to now further develop those [plans for collaboration]…going back to the government and saying “this is likely to be the option; now give us more information,” and get also some commitment from the government for those particular options…So we are at a point where now we are developing detailed work plans and budgets around this and also getting commitment from government and other partners* (AHME Implementing Partner, Kenya).

Rather than approaching the NHIs by prescribing plans of action, the collaboration built on mutual problem-solving and the AHME partners working with continuous feedback from NHI partners. This process of designing and implementing pro-poor programmes allowed both parties to learn about their respective communication styles, which in turn allowed them to work together more smoothly.

When the programmes moved past the design phase, it remained critical for the AHME partners to meet regularly with the NHIs. Thus, the development of formalized arenas for meeting, such as the HISP technical working group in Kenya, was essential to furthering the success of their collaboration. Meeting formally, in-person, and on a regular basis both strengthened the relationship and created avenues for the AHME partners to open up discussions about other areas of interest and to influence the public sector’s view of private providers:

***AHME has created space*** *, and so we can go beyond what they have been doing, engaging different units of the government, and then starting to talk about other aspects which the public sector is not aware of, and they become interested in the private sector* (AHME Implementing Partner, Kenya).

### Shared value in collaboration through AHME

Interviews with the AHME partners and with government officials suggested that mutual suspicion, or at least the partners’ perception of mutual suspicion, has waned since AHME began and a more ‘symbiotic’ relationship has developed. Several implementers specifically reported noticing a change in how they work with government over the course of the AHME project; they believe that NHI representatives now view private providers as partners more so than in the past. As one interviewee described:

*There is more willingness on the part of the government to want to engage more actively with the private sector and even willing to take some level of risk* (AHME Implementing Partner, Ghana).

Further, one NHIF official in Kenya noted that partnering with the private sector has become a point of pride for government:

*Working with the different NGOs and institutions has been a plus for NHIF and it goes even beyond us. When we are doing the scale up [of the HISP program], targeting more beneficiaries country wide, increasing the numbers*, ***we will be able to say that it was not just a public institution affair, it was a public-private partnership****. We were able to collaborate at various aspects of the projects so going forward it would propagate the need for more private-public partnerships, even in the country* (NHIF Official, Kenya).

Unlike the suspicion and mistrust that the AHME partners perceived in the early rounds of the evaluation, NHI officials see how public–private collaboration with small private providers can allow them to make more progress towards their goals now and in the future. Further, AHME has not only influenced government’s willingness to collaborate with the partnership, but partners perceived a shift in government views of the private sector more broadly, particularly smaller clinics that were unattractive to the public sector in the past.

*For a long time, the government never really used to empanel private sector facilities especially of our level. They were more interested in [large private hospitals]…But now you have these small providers…they were not attractive at all…We’ve changed that. So now they see the smaller facilities from a different lens* (AHME Implementing Partner, Kenya).

An important benefit of the relationship between AHME and government partners has been the development of a feedback loop, whereby the AHME partners act as an intermediary to communicate private provider experiences directly to the NHIs. This feedback loop is mutually beneficial; on the one hand, the AHME partners act as communication channels from the NHIs back down to the ground, keeping providers informed about government policies and helping them navigate challenging bureaucracy.

*But now increasingly they have seen the value of this partnership. What we’ve seen is…now we can be able to influence [policy]…This is the feedback we are hearing from the providers and then they channel back to the branches and the branches act on it.* ***So it’s been a very symbiotic relationship, and it’s a journey but we are proud of it*** (AHME Implementing Partner Kenya).

On the other hand, the feedback loop is a conduit where the practices and challenges of private providers are channelled back up to the public sector. As one NHIF official noted:

*We were able to sit as a team because we were now with PharmAccess, PS-Kenya and MS-Kenya most of it now on the ground because that’s where now the communication strategy was being implemented and we were able to work out the small issues…Now, here we are with MS-Kenya who have that person in the village or they have that clinic down in the village or PS-Kenya who has that community health worker who works with that beneficiary welfare committee person.* ***So with time we were able to be able to know how to utilize those people*** (NHIF Official, Kenya).

Several concrete changes have developed as a result of this cycle of feedback. In Kenya, e.g. feedback from the AHME implementing partners influenced a change in the licensing required for NHIF accreditation, which made accreditation cheaper and more accessible for small private providers. In addition, the NHIF streamlined their accreditation process, temporarily replacing the in-person inspection requirement with a self-administered checklist.

## Discussion

The AHME example illustrates the importance of shared goals, mutual understanding, and structured, ongoing communication to develop a strong working relationship between the public and private sectors when collaborating around health financing. Although public–private collaboration was largely driven by the AHME partners from the private sector side, the partnership was mutually beneficial. The partners gained traction with their public-sector counterparts because the goals of the partnership aligned with government goals, and AHME offered the resources and expertise that the public sector lacked to achieve these goals. Further, the partnership as a whole established the connection with government through partners that already had a relationship with the NHIs (specifically, the IFC), which allowed other partners to get a foot in the door. Once these connections had been made, all of the partners were able to develop working relationships with the NHIs over time through structured and sustained interactions, resulting in increased understanding of the value of public–private collaboration. As these relationships grew, they created feedback loops between NHI bureaucrats and private providers on the ground, ultimately giving the providers more voice in the system.

Our findings suggest some key factors to consider when designing a public–private partnership around health financing, and also align with some of the previous work on PPPs in Ghana and Kenya (Hill *et al.* 2016; Hushie 2016). Specifically, we found that the partnership between AHME and the NHIs addressed some of the gaps identified by Ravishankar *et al.* (2016), such as a lack of communication that results in mistrust and a general lack of engagement between government and private providers that impedes private provider understanding of government policies and procedures. However, our findings also point to some challenges. As noted in an earlier study in Ghana (Amo-Adjei 2016), co-ordinating across sectors, particularly with a number of partners involved across the globe, was sometimes slow and inefficient due to multiple bureaucracies interacting. Developing protocols for interaction and communication across sectors could make this coordination smoother and more efficient. This can be done by formalizing policy arrangements such that private sector representatives are regularly involved in policy design and all parties use standardized processes and templates when working together (Ravishankar *et al.* 2016), creating legally binding agreements, or somewhat less formally by maximizing opportunities for interaction and creating an organizational culture of openness and sharing across sectors (Brinkerhoff 2003). However, clear organization among the partners representing private providers, with defined roles and lines of communication, will also be key to increasing efficiency in cross-sector work. This may be a valuable area for further inquiry, as much of the literature on NGO partnerships focuses either on PPPs or on partnerships between NGOs in the Global North and those based in the Global South (Corbin *et al.* 2013; Contu and Girei 2014), as opposed to collaborations among a group of INGOs with equal standing.

Consistent with prior research (Sieverding *et al.* 2018), our findings also suggest that social franchise networks can be an effective avenue to engage private providers in the health system. The franchise networks supported by the AHME partners offer an opportunity for the public and private sectors to partner in a new and more efficient way. However, private providers outside of franchise networks may warrant additional research regarding their experiences working with the public sector. Since independent private sector providers are much less organized than those in social franchise networks, they may face unique challenges to meaningful integration into the health system.

Finally, as the NHIs and the private sector continue to develop their relationship, both AHME partners and NHI officials suggested that the future of PPPs for health financing, and partnerships with social franchise networks in particular, will focus on engaging with networks as a bundle rather than with individual providers. With this vision in mind, the AHME partnership has plans to pilot models that aggregate franchised providers to work with the NHIs as a single group. Theoretically, these efforts have the potential to make the NHI accreditation process more efficient for both government and providers, and ultimately to bring Kenya and Ghana closer to UHC by improving provider reach. However, to our knowledge this model has not been studied extensively and largely in developed countries (Bazzoli *et al.* 2000; Draper *et al.* 2007; O’Connor and Spector 2014). So, the specific challenges of working within LMIC health systems are not yet known, although testing these models poses a unique and potentially valuable learning opportunity.

## Conclusion

Although PPPs have become more popular in the healthcare field and may enhance the reach of health systems in LMICs to achieve UHC, we know little about how these partnerships work above ground level. This case study of collaborations between a group of INGO partners and the NHIs in Ghana and Kenya indicates that shared goals, mutual understanding of what each sector can offer in a partnership; and ongoing, structured communication are key factors in creating a successful partnership. However, developing joint protocols for communication as well as internal protocols among the INGOs will be important for sustaining successes and creating avenues for future work together. Aggregating franchised private providers to facilitate their interactions with government may be one way to encourage ongoing partnership, although little is known about implementing this model in LMICs at this time.

## Ethical considerations

The ethical review boards of the University of California San Francisco, Ghana Health Services, and Kenya Medical Research Institute approved the study protocols in each round of data collection. The research team obtained consent from all interviewees.

## Footnotes

1 Social franchising applies the principles of commercial franchising to health services with the goal of building a network of providers that deliver a minimum standard of quality. Each franchised clinic is individually owned and contracted to a common agency (usually an NGO) that provides standardized, often subsidized, products and services under a common brand in addition to opportunities for training. The franchisors typically monitor clinic performance and require members to maintain minimum levels of performance in addition to paying regular membership fees (Schlein and Montagu 2012).

2 In the design phase of the AHME project, partners were selected by the funding organizations for their unique strengths and expertise, including: service delivery (MSI and PSI); quality assurance and service expansion (PharmAccess Foundation); and enabling a friendly policy and regulatory environment (IFC).

3 Interviewees are drawn from one of three groups: Global Partners are drawn from the donor organizations (Bill and Melinda Gates Foundation and the UK Department for International Development) as well as leadership within MSI, since that organization was designated as the lead within the AHME project. In-Country Partners are involved directly in implementation of AHME objectives, and are members of the country teams of MSI, PSI, PA or IFC. Government Representatives are employed by either the NHIA or National Health Insurance Fund (NHIF) and have directly collaborated with the AHME partners.

4 Note that the sample size of NHIF officials interviewed in 2014 was small (*n* =2) and no NHIA officials in Ghana were interviewed in this early round of data collection.
