# Public–private partnerships in practice: collaborating to improve health finance policy in Ghana and Kenya

**DOI:** 10.1093/heapol/czy064

**Published:** 2018-07-20

**Authors:** Lauren Suchman, Beth Ann Hart, Dominic Montagu

*Health Policy and Planning*, doi: 10.1093/heapol/czy053.

The authors wish to apologize for the following error: the name of one of the co-authors, Beth Ann Hart, was incorrect as initially provided. This has now been corrected in print and online.

